# Intra‐ and interhost genomic diversity of monkeypox virus

**DOI:** 10.1002/jmv.29029

**Published:** 2023-08-11

**Authors:** Mona L. Taouk, Eike Steinig, George Taiaroa, Ivana Savic, Thomas Tran, Nasra Higgins, Stephanie Tran, Alvin Lee, Maxwell Braddick, Michael A. Moso, Eric P. F. Chow, Christopher K. Fairley, Janet Towns, Marcus Y. Chen, Leon Caly, Chuan K. Lim, Deborah A. Williamson

**Affiliations:** ^1^ Department of Infectious Diseases The University of Melbourne at the Peter Doherty Institute for Infection and Immunity Melbourne Victoria Australia; ^2^ Victorian Infectious Diseases Reference Laboratory The Royal Melbourne Hospital at The Peter Doherty Institute for Infection and Immunity Melbourne Victoria Australia; ^3^ Victorian Department of Health Melbourne Victoria Australia; ^4^ Melbourne Sexual Health Centre Alfred Health Melbourne Victoria Australia; ^5^ Central Clinical School, Faculty of Medicine, Nursing and Health Sciences Monash University Melbourne Victoria Australia; ^6^ Centre for Epidemiology and Biostatistics, Melbourne School of Population and Global Health The University of Melbourne Melbourne Victoria Australia

**Keywords:** monkeypox virus, mpox, whole genome sequencing

## Abstract

The impact and frequency of infectious disease outbreaks demonstrate the need for timely genomic surveillance to inform public health responses. In the largest known outbreak of mpox, genomic surveillance efforts have primarily focused on high‐incidence nations in Europe and the Americas, with a paucity of data from South‐East Asia and the Western Pacific. Here we analyzed 102 monkeypox virus (MPXV) genomes sampled from 56 individuals in Melbourne, Australia. All genomes fell within the 2022 MPXV outbreak lineage (B.1), with likely onward local transmission detected. We observed within‐host diversity and instances of co‐infection, and highlight further examples of structural variation and apolipoprotein B editing complex‐driven micro‐evolution in the current MPXV outbreak. Updating our understanding of MPXV emergence and diversification will inform public health measures and enable monitoring of the virus’ evolutionary trajectory throughout the mpox outbreak.

## 1 INTRODUCTION

Mpox is a viral zoonotic disease caused by monkeypox virus (MPXV) belonging to the *Orthopoxvirus* genus, previously endemic to West and Central Africa.^1^ A multicountry outbreak of MPXV was confirmed in May 2022 and was declared a public health emergency of international concern by the World Health Organization (WHO) in July 2022, marking the first time mpox has spread widely outside of traditionally endemic areas.^2^, ^3^, ^4^, ^5^, ^6^, ^7^ As of April 2023, there have been more than 86 000 confirmed cases globally during this outbreak, primarily reported from Europe and the Americas, although encompassing more than 110 countries globally.^8^, ^9^

Genomic analysis has played a role in the ongoing mpox outbreak, including the tracking of mpox spread and diversification, and importantly, identifying the emergence of lineages, which may have clinically relevant phenotypes.^10^, ^11^, ^12^ Clades, lineages, and sublineages have been defined from publicly available sequence data and are updated regularly as MPXV continues to diverge.^13^, ^14^ Genomic sequencing has demonstrated two major clades of MPXV, with the current outbreak forming a distinct lineage (Clade IIb) within Clade II, although co‐circulating lineages within Clade II have been identified in the Northern Hemisphere Among these, the vast majority have been associated with the B.1 lineage of Clade IIb.^10^ Genomic studies have also implicated the host apolipoprotein B editing complex (APOBEC3) cytosine deaminase as a driver of ongoing MPXV evolution and human adaptation.^15^

During the COVID‐19 pandemic, genomic surveillance directly informed public health responses. Severe acute respiratory syndrome coronavirus 2 (SARS‐CoV‐2) genomic diversity, coupled with epidemiological data, enabled identification of transmission clusters, and in some settings, directly informed contact tracing efforts.^16^ To date, there are limited data on the utility of genomic data for assessing local transmission of mpox. Recent studies suggest a degree of intrapatient diversity of MPXV genomes, with apparent genomic variation both within and between anatomical sites of infection.^5^, ^11^, ^17^ This potential intrapatient genomic diversity, coupled with the slow mutation rate of orthopoxviruses (estimated at 1–2 single‐nucleotide polymorphisms [SNPs] per year), may make accurate inferences of interpatient transmission challenging, and requires rigorous validation of the variants observed in the current outbreak strains.^18^

In Australia, mpox is a nationally notifiable disease, with 144 cases (confirmed and probable) diagnosed in Australia as of May 4, 2023.^8^ Unlike other parts of Australia, most cases of mpox in the state of Victoria are epidemiologically linked to local transmission, rather than incursions from overseas. Here, to better understand the utility of genomic data for informing public health responses we undertook sequencing of MPXV in Victoria. In this study, we describe the genetic diversity of the B.1 outbreak of mpox in Victoria, Australia. Our findings reveal evidence of multiple introductions of mpox, followed by sustained local transmission. Additionally, our analysis suggests the presence of within‐host microevolution, which may be differentiated from co‐infection with multiple strains. These findings underscore the significance of assessing within‐host variation in future studies concerning the evolution and transmission of mpox.

## 2 RESULTS

### 2.1 MPXV genomes reveal multiple introductions of mpox into Australia

To better understand the utility of genomic data for informing public health responses, we performed whole genome sequencing (WGS) on all positive MPXV samples collected between May 2022 and September 2022 in Victoria, Australia. In total, 102 genomes from 56 individuals, were successfully sequenced and included in analyses (Supporting Information: Appendix Figure 1). The median number of samples sequenced per patient was two, ranging from one to seven (Supporting Information: Figure 2). All individuals were male and the median age at specimen collection was 37 years (interquartile range of 30.0 to 41.3). Available epidemiological information suggested that 50.0% (28/56) of individuals acquired their MPXV infection within Australia, whereas 39.2% (22/56) acquired their infection overseas; for a subset of individuals (10.7%; 6/56), the location of infection was unknown. Cycle threshold (*C*_t_) values were used to approximate viral load from these samples, with *C*_t_ values being significantly lower (*p* < 0.0001) in samples from lesions (skin, anorectal, and genital), compared with oral, nasopharyngeal, or other (blood, urine) samples (Supporting Information: Figure 2).^19^ There was also a significant correlation (*R* = 0.67, *p* < 0.0001) between sample *C*_t_ value and consensus genome completeness relating to that sample (Supporting Information: Figure 2).

**Figure 1:**
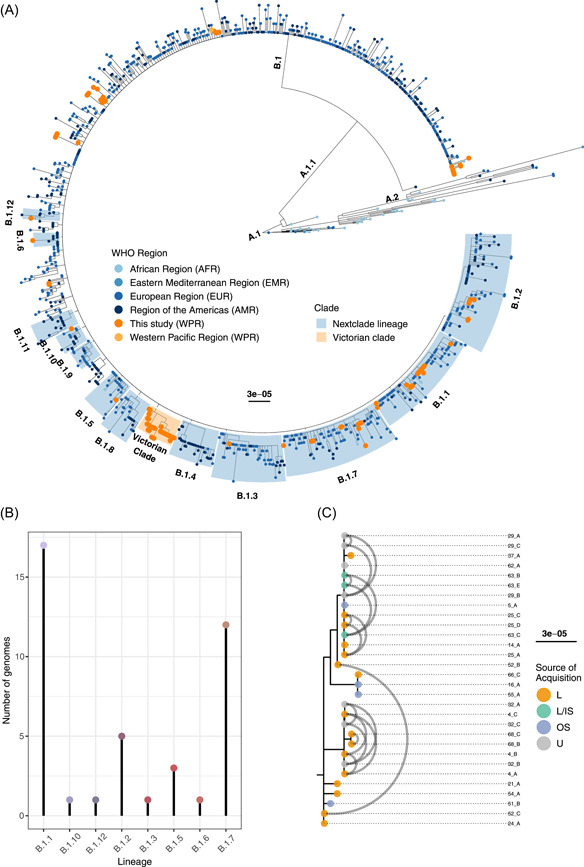
Phylogenetic structure of monkeypox virus (MPXV) genomes included in this study. (A) A midpoint rooted maximum likelihood phylogeny showing the population structure of 1105 MPX genomes included in this study. Clades are highlighted to show lineages (blue) and a group of interest (yellow). Tips are annotated to show the World Health Organization (WHO) region where each genome was collected. Samples collected in this study are colored yellow. Clades are labeled on their branches. The tree scale representes substitutions per site. (B) The number of genomes sequenced in this study belonging to each lineage. This does not including the ancestral B.1 lineage that all genomes sequenced in this study belong to. (C) A zoomed‐in clade showing the Victorian group of interest, with tips colored by suspected location of mpox infection and gray arcs connecting genomes collected from the same patient. The tree scale representes substitutions per site. IS, interstate; L, local (Victoria); OS, overseas; U, unknown.

To assess the relatedness between genomes of MPXV in Victoria and those circulating internationally, we generated a maximum likelihood (ML) phylogeny. Following exclusion according to quality control criteria, 1105 genomes were included in this phylogeny (102 Victorian and 1003 publicly available) (Supporting Information: Data set 1). Applying recently described lineage designations,^13^ all 102 Australian MPXV genomes fell within the 2022 outbreak lineage (B.1), of MPXV Clade IIb (Figure 1A). Victorian samples were distributed throughout the phylogeny and within multiple lineages of Clade IIb, indicative of multiple incursions of MPXV into Australia (Figure 1B). One sample (64_B) was defined as B.1 but clustered within the B.1.6 clade, sharing an ancestor with the other B.1.6 samples. Upon manual inspection, the region containing the B.1.6 lineage defining SNP (G111046A) was called as an N nucleotide, causing this misclassification.

### 2.2 Phylogenetic analysis of MPX genomes is suggestive of local transmission

Based on the ML phylogeny, some genomic features indicative of sustained local transmission were noted. For example, within lineage B.1, we identified a large monophyletic group consisting of 30 Victorian samples, collected from 18 individuals (Figure 1B). Available epidemiological information suggested a subset (22.2%; 4/18) of individuals in this group acquired their infections overseas, with multiple continents implicated (Figure 1B), whereas the majority acquired their infections in Victoria, Australia (10/18; 55.6%), and one individual was thought to have acquired mpox from another state in Australia (1/18; 5.6%) (Figure 1B). All 30 genomes in this clade shared the same two SNPs: G53343A and C121121T (Figure 1B), suggesting these variants were present in the common ancestor of the group. Although no international samples shared both SNPs simultaneously, we identified G53343A in eight publicly available genomes (GenBank accessions: OP442549, OP442551, OP526857, OP539924, OP615274, OP680491, OP680497, and OP687932). Conversely, C121121T was not observed in any publicly available genomes outside of the Australian clade.

Nextstrain maintains a list of lineage defining SNPs used to characterize MPXV genomes as belonging to one of 14 lineages (B.1.1–B.1.14). However, a subset of Victorian and publicly available genomes had a lineage defining variant but did not fall within the lineage clade phylogenetically (Supporting Information: Appendix). As of January 30, 2023, the majority (94.4%; 17/18) of lineage defining SNPs occured within a coding sequence, with 76.5% (13/17) resulting in a nonsynonymous coding change and 94.4% (17/18) being putatively derived from APOBEC3 activity (Table 1). Further, there was evidence of genomes containing a lineage defining variant for more than one lineage. For example, in the Victorian data set, two genomes (34_A and 34_B) had the B.1.5 lineage defining SNP (C70797T) as well as the B.1.2 lineage defining SNP (G186144A), but were clustered in the B.1.2 clade of the phylogeny (Supporting Information: Appendix and Figure 4).

**Table 1:** Gene deletion.

Individual	Gene (ON562414.3)	Vaccinia virus homolog	Description	Gene size (bp)^a^	Deletion type
34	*MPXVgp170*	*B8R*	Soluble interferon‐γ receptor‐like protein	804	Partial
34	*MPXVgp171*	*B9R*	Intracellular viral protein	666	Full
34	*MPXVgp172*	*B12R*	Ser/thr kinase	849	Partial
34	*Hypothetical CDS*	*B11R*		303	Full
48	*MPXVgp023*	*N1L*	Bcl‐2‐like protein	354	Partial
48	*MPXVgp024*	*N2L*	Bcl‐2‐like protein	534	Full
48	*MPXVgp025*	*M1L*	Ankyrin‐like protein	1,329	Full
48	*MPXVgp026*	*M2L*	NFkB inhibitor	663	Full
48	*MPXVgp027*	*K1L*	Ankyrin‐like protein	855	Full
48	*MPXVgp028*	*K2L*	Serpin	1,128	Partial

a Gene size refers to the number of base pairs in the open reading frame of the gene and does not include upstream or downstream noncoding regions. Deletion type refers to the amount of gene deleted where “full” means the whole gene is deleted and “partial” means some of the gene was deleted.

### 2.3 Characteristics of interhost genomic diversity

**Figure 2:**
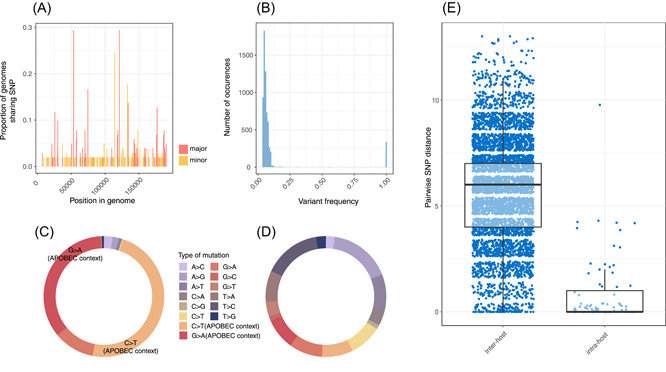
Inter‐ and intrahost minor and major variants. (A) The proportion of major and minor single‐nucleotide polymorphisms (SNPs) shared between more than one genome for each unique position in the genome. Major variants are defined as those present at ≥75% frequency and minor variants are defined as those variants present at a frequency between 5% and 75%. In all samples, major and minor variants were not considered in repeat regions and minor variants were only considered in sequenced on an Illumina platform. (B) The distribution of variant frequencies across both major and minor SNPs. (C) The proportion of major SNPs by each type of mutation across all genomes. (D) The proportion of minor SNPs by each type of mutation across all genomes. (E) The pairwise SNP distance between genomes collected from the same host or different hosts. Box plots indicate median and interquartile range (IQR), with the whiskers representing the highest and lowest values within 1.5 × IQR of the upper and lower quartiles, and the dots representing each pairwise SNP distance between any two genomes from the same or different individuals.

Overall, we observed low genetic diversity among Australian sequences; particularly when only considering major SNPs (≥0.75 variant frequency). A total of 97 unique major SNPs were identified in nonrepeat regions relative to the B.1 reference (MPV_USA_2022_MA001, GenBank ON563414.3), with a median of three SNPs per genome (range of 0–8) (Supporting Information: Figure 3 and Data set 2). Previous reports have described variation in allele frequencies within a patient, suggesting the existence of intrahost diversity where emerging variants are yet to become fixed.^11^ As such, exploration of minor SNPs (0.05–0.74 variant frequency) in Illumina samples from this study was undertaken. For samples sequenced on an Illumina platform, 5699 unique minor SNPs were detected in nonrepeat regions, with only 182/5699 (3.2%) of these shared between more than one genome (Figure 2A and Supporting Information: Figure 3 and Data set 2). Contrary to previous reports, major SNPs were almost all fixed, with very few (314/5699; 5.5%) occuring at a frequency between 10% and 75% in a single sample (Figure 2B). There was an abundance of GA to AA (34/97, 35.1%) and TC to TT (46/97, 47.4%) major SNPs, indicative of APOBEC3 activity (Figure 2C). However, minor SNPs were distributed more equally between mutation profiles (Figure 2D). There were no major SNPs in MPXVgp083, a palmitoylated EEV membrane protein homologous to F13L in vaccinia virus and the putative target of the therapeutic antiviral tecovirimat currently used to treat mpox.^20^

### 2.4 Characteristics of intrahost genomic diversity

**Figure 3:**
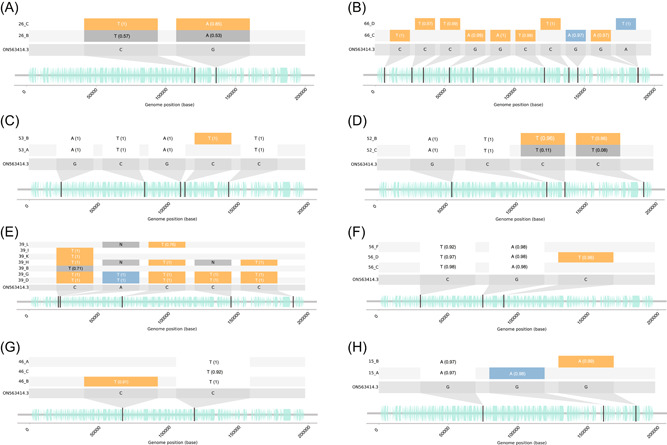
Single‐nucleotide polymorphisms (SNPs) relative to the US reference genomes for the eight individuals whose samples displayed intrahost variation. Positions and reference nucleotides are relative to the US B.1 reference genome (MPV_USA_2022_MA001, GenBank ON563414.3) and shown on the X axis including positions and directions of genes. Rectangles representing variants that are conserved across genomes from the same individual are shaded by a white square; rectangles representing variants that are variable between genomes from the same individual are colored yellow; for variants that are targets of APOBEC3 are colored blue for all other variants. The major variant frequency is shown next to each SNP in brackets, as a proportion of variant reads/total reads. Where available, the minor variant frequency is shown in a gray rectangle, as a proportion of variant reads/total reads. Genomes from panels A, C–E, and G were sequenced on Illumina devices, whereas genomes from panels B, F, and H were sequences on Oxford Nanopore Technology devices.

Establishing the diversity within a patient or between epidemiologically linked pairs can be informative in understanding viral evolutionary dynamics.^21^ In our study, there were 27 individuals (27/56; 48.2%) for whom multiple samples had been sequenced, either from more than one body site or as longitudinal sampling. There was a median pairwise SNP distance of six SNPs (range of 0 to 14) between genomes collected in Australia from different individuals, including 146 pairs of identical genomes between 34 different individuals. 16/34 (47.1%) of individuals reported acquiring their infection within Victoria, and a further 2/34 (5.9%) reported acquiring their infection interstate, suggesting potential community transmission. In comparison, the median pairwise SNP distance between previously published B.1 genomes was five (range of 0 to 108). Although the median pairwise SNP distance between genomes collected from the same patient was zero, we observed 8/27 (29.6%) individuals with one to 10 SNPs across different genomes (Figure 2E). By analyzing the frequency of variants from MPXV genomes within individuals, we estimate that any two randomly chosen genomes collected from a single individual differ by a mean of 0.77 variants. Of variant sites that have potentially evolved within an individual, 20/24 (83.3%) were indicative of APOBEC activity (GA to AA: 5/20, 25.0%; and TC to TT: 15/20, 75.0%) (Figure 3A–D).

To better understand whether these samples represented co‐infection or intrahost diversification, we undertook a fine scale analysis of samples from the eight individuals, which showed MPXV diversity (Figure 3A–E). In some individuals, it was possible that intrahost variation may represent intrahost evolution. For example, in a genital sample from one individual (sample 26_C), the C118438T SNP was observed at a variant frequency of 1. However, in a skin sample from the same individual (sample 26_B) this SNP had a minor variant frequency of 0.57 (Figure 3A and Supporting Information: Data set 2). Similarly, in sample 26_C, the C133816T SNP was present at a major variant frequency of 0.85, whereas only present in 26_B at a minor variant frequency of 0.53, suggesting potential within‐host evolution. A similar relationship was observed between genomes from another individual (53_A, and 53_B; Figure 3C and Supporting Information: Data set 2). Alternatively, other patterns of intra‐host variation may represent co‐infection. For example, one individual reported acquiring their infection in Victoria and had two samples collected on the same day from a face lesion (66_C) and a perianal lesion (66_D). The resulting genomes were separated by 10 pairwise SNP differences, sharing no variants against the reference (Figure 3B and Supporting Information: Data set 2). The genomes occupied distant positions in the phylogeny, with 66_C identical to two other Victorian genomes (16_A and 55_A) and positioned within the monophyletic Victorian group, and 66_D having branched off from the B.1 lineage independently, suggesting that this patient may have been infected with two independent viruses concurrently. A similar pattern was observed in genomes from another individual (15_A, 15_B; Figure 3H and Supporting Information: Data set 2). However, in 2/8 individuals, it was unclear whether intra‐host variation was due to evolution, a co‐infection, or another mechanism (Figure 3D–G).

### 2.5 Diversity in Victorian MPXV genomes include large‐scale multigene deletions

**Figure 4:**
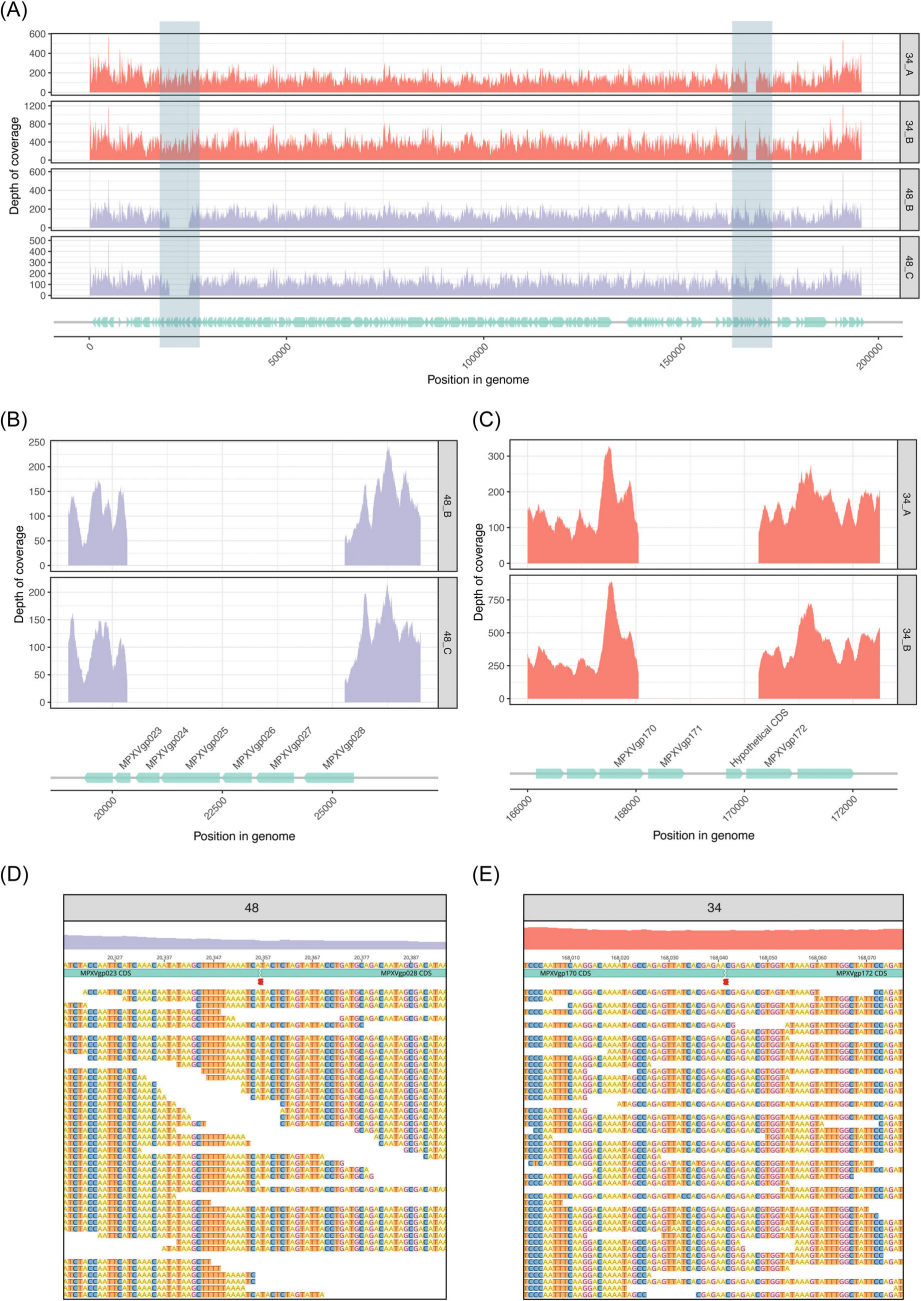
Regions of observed deletions. (A) Depth of sample reads against the US B.1 reference genome (MPV_USA_2022_MA001, GenBank ON563414.3) with position (base pairs) in genome on the X axis including positions and directions of genes. Coverage, or number of aligned reads, are plotted on the Y axis. The two regions of deletions are highlighted by gray rectangles. (B) The coverage of reads from 48_B and 48_C at the site of deletion with genes involved in the deletion labeled. (C) The coverage of reads from 34_A and 34_B at the site of deletion with genes affected by the deletion labeled. (D) Sample 48 reads mapping to an edited version of the US B.1 reference genome with the region from nucleotide positions 20 358–25 294 removed. (E) Sample 34 reads mapping to an edited version of the US B.1 reference genome with the region from nucleotide positions 168 042–170 251 removed.

In addition to APOBEC3 activity, the large‐scale deletion and rearrangement of MPXV genetic material is another putative feature of MPXV diversification and evolution, and was observed in this data set (Supporting Information: Appendix and Figure 5).^22^, ^23^ Large‐scale genetic deletions were observed for four genomes collected from two individuals (34_A, 34_B, 48_B, and 48_C) (Figure 4A). In one individual, a 4937 bp deletion in the 5′‐end of the genome was confirmed (nucleotide positions 20 358–25 294) (Figure 4B). Similarly, in another individual, we observed a 2210 bp deletion in the 3′‐end of the genome (nucleotide positions 168 042–170 251) (Figure 4C). It was found that no reads aligned to these regions for each respective patient, and the boundaries of the deletion were confirmed through further read alignment and Sanger sequencing, indicating large deletions in the genome (Figure 4D,E). The deletion in individual 34 affected four genes and the deletion in individual 48 affected eight genes (Table 1). Both individuals were thought to have acquired their infection in Australia; however, no other samples displayed either deletion profile, suggesting there was no onward transmission of these strains.

## 3 DISCUSSION

In this study, we examined the emergence and spread of mpox in Victoria, Australia, during 2022, which marked the first introductions of the virus into the country. Our data highlight incursions of multiple lineages, with subsequent local transmission in some instances.

All MPXV genomes generated in this study belonged to the B.1 lineage of Clade IIb, sharing a recent common ancestor with sequences from Nigeria.^10^ Although we describe limited diversity between genomes, robust phylogenetic structure was noted including many previously defined lineages, with the most common lineage being B.1.1 (17/102 genomes; 16.7%). In addition, a clade specific to Victoria was identified, comprising 30 genomes collected from 18 individuals. Two SNPs (G53343A and C121121T) were shared between these 30 genomes, with C121121T not observed in any other publicly available genomes (as of January 30, 2023). This may suggest community transmission in Victoria, consistent with epidemiological data suggesting that 10/18 (55.6%) of these individuals acquired mpox in Victoria. This clade may therefore represent a MPXV lineage circulating internationally, which has not yet been identified due to an underrepresentation of global genomes in public databases. To date, most sequences have been collected from the WHO Americas Region, with very few sequenced genomes collected from the WHO Western Pacific Region, and only one previously shared genome from Australia.^24^ As for other infectious diseases, genomic surveillance is optimal when there is consistent sampling and data sharing from all relevant geographic areas.^25^, ^26^ Further sampling and sequencing within the Western Pacific Region is essential to better understand MPXV genetic diversity present in this region.

We also observed that the currently maintained lineage‐defining SNPs (https://github.com/nextstrain/monkeypox) may not be phylogenetically stable, as may be expected in the relatively early stages of an outbreak. For example, we identified instances of Victorian and publicly available genomes having a lineage‐defining SNP but being placed outside of the lineage‐specific clade in the phylogeny, or genomes containing the lineage defining variant for more than one lineage. This may be due to a significant proportion of SNPs in the genome occurring as a result of the nonrandom mutational driver, APOBEC3, causing coding changes in genes under selective pressure such as surface proteins and resulting in convergent evolution. To be stable, typing schemes should ideally be based on synonymous SNPs in essential genes, occurring in every member of a lineage and not occurring outside of the lineage.^27^ As further variation emerges and lineage defining schemes are updated, a preference could be given for inclusion of intergenic and synonymous variants to address possible convergent evolution.

Our observation of low MPXV genetic diversity align with other studies, particularly among the B.1 lineage of Clade IIb.^10^, ^11^ We found that most major SNPs (80/97; 82%) likely occurred as a result of APOBEC3 activity, consistent with previous findings.^11^, ^15^ Further, contrary to previous reports, we observed a bimodal distribution of SNP frequencies, with very few (5.5%; 314/5699) SNPs occurring at an intermediate frequency between 10% and 75%.^11^ The observation that fixed variants were commonly APOBEC‐driven, whereas minor variants did not share this pattern, may suggest a selective advantage for GA > AA and/or TC > TT variation by way of adaptive codon usage or GC content, as described for other viruses.^28^ The limited genetic diversity of MPXV makes it challenging to use sequence data alone for inter‐patient transmission analyses or fine‐resolution contact tracing of outbreaks, unlike the use of genomics for SARS‐CoV‐2 transmission.^16^ In the early stages of an outbreak, the causative pathogen may not have diverged sufficiently to confidently use sequence data to infer chains of transmission.^29^ In this context, genomic surveillance of MPXV may be better utilized at present in understanding viral evolution, and early identification of mutations associated with vaccine or diagnostic escape, or antiviral resistance.^30^

In contrast to the limited utility of genomic analysis for transmission analysis, we found considerable genetic diversity within individual mpox infections, with an expected 0.77 SNPs between any two genomes collected from a single individual at different body sites on the same day. Additionally, we described at least two instances where patterns of intrahost diversity, including the proportion of shared variants against the reference genome and minor variant frequencies, indicated potential intra‐host evolution. Based on an average incubation period and time from symptom onset to sample collection of mpox of ~3 weeks, and the rate of evolution of MPXV estimated at 0.68 SNPs per 3 weeks (https://github.com/nextstrain/monkeypox), these findings are broadly congruent with the hypothesis that intrahost variation is due to the accumulation of de novo mutations within the host.^1^ The clear majority of our samples from a single patient appeared to comprise a single lineage or share a phylogenetic clade. Putative co‐infection was observed in two individuals, and in one of these individuals, two genomes collected from different anatomical sites were separated by 10 variants—far beyond what could be expected as resulting from intrapatient evolution alone. The analysis of intrahost variants have been used in conjunction with other phylogenetic methods to improve the inference of interhost transmission chains in other viruses and bacteria including SARS‐CoV‐2, which could be applied to MPXV going forward.^21^, ^31^, ^32^

Gene loss, duplication, and rearrangements have been documented in orthopoxviruses and may be associated with viral pathogenicity and host range.^33^, ^34^, ^35^ These are most common in the genomic termini, where non‐essential and virulence genes are housed.^36^ It has been suggested that gene loss may serve as a mechanism for orthopoxviruses to evolve or adapt to a new host.^37^ Such mutations may indicate that MPXV is adapting to human‐to‐human transmission. In our data set, we found large‐scale genomic deletions in the termini of four genomes, from two individuals. Our findings are consistent with the few other reports describing large‐scale rearrangements in the MPXV genome, including deletions.^7^, ^38^, ^39^ It is currently unclear if these large‐scale mutations are adaptive, neutral, or disadvantageous for the virus, with future work needed to extrapolate these mutations to phenotypic fitness or clinical presentations. The deletions described in this study do not include targets for frontline diagnostic assays; however, in line with previous studies, they do indicate that there is a continued need for genomic surveillance of MPXV to study its evolution during this outbreak to ensure current diagnostic approaches and medical treatments remain effective.^40^

Although our study in Victoria, Australia, identifies multiple globally present lineages of MPXV, as well as showing local transmission, the low genetic diversity of MPXV poses challenges for transmission analysis using sequence data alone. Further research is needed to understand MPXV genetic diversity and evolution in the Western Pacific Region, including potential intra‐host evolution and gene changes associated with pathogenicity and host range. These findings contribute to our understanding of the genomic characteristics and evolution of MPXV in the context of its trajectory in Victoria, Australia.

## 4 METHODS

### 4.1 Setting and data sources

Clinical samples sent for MPXV polymerase chain reaction (PCR) testing between May 19, 2022 and September 7, 2022 were obtained from the Victorian Infectious Diseases Reference Laboratory (VIDRL) at The Peter Doherty Institute for Infection and Immunity (Melbourne, Australia). VIDRL is the public health virology laboratory for the state of Victoria, serving a population of ~6.24 million people. Clinical samples were obtained from various testing sites in Victoria and stored in Universal Transport Medium (Copan, Italy) at −80°C. Sample types included anorectal, genital, oral/nasopharyngeal and skin lesion specimens. Deidentified information on suspected place of acquisition was provided by the Victorian Department of Health. Place of acquisition was defined as local (acquired in the State of Victoria), interstate (acquired in Australia outside of Victoria) or overseas (acquired outside of Australia). Ethical approval was obtained from the Alfred Hospital Ethics Committee (project 505/22).

### 4.2 Genomic DNA extraction, real‐time PCR, and sequencing

Genomic DNA was extracted from clinical samples using the QIAcube HT automated machine with the QIAamp 96 Virus QIAcube HT kit according to manufacturer's instructions. WGS was attempted on residual clinical specimens that were positive for MPXV by PCR at VIDRL, based on a previously described in‐house real‐time PCR.^24^ For 80 samples, WGS was performed on an Illumina iSeq. 100 instrument (Illumina), with 150 bp pair‐end reads using the “Twist Total Nucleic Acids Library Preparation Kit for Viral Pathogen Detection and Characterization” for Library prep and the “Twist Target Enrichment Standard Hybridization v1 Protocol” using the Comprehensive Viral research panel for enrichment (Twist Bioscience). For 115 samples (Supporting Information: Data set 1), library preparation was performed on extracted genomic DNA using an adapted version of the protocol published by Oxford Nanopore Technologies using the Rapid Barcoding Kit 96 and Midnight RT PCR Expansion for barcoding and library preparation primers to generate 2500 bp amplicons.^41^ WGS was performed on an GridION sequencer (Oxford Nanopore Technologies), using a R9.3.1 Flow cell (Oxford Nanopore). Basecalling was performed using guppy (v.6.1.5) on GridION using the high‐accuracy models.

### 4.3 Bioinformatic analysis

For samples with Illumina reads only, read quality control was assessed using Fastp v0.23.2 (default settings with: ‐‐length_required 50 –cut_tail –cut_tail_mean_quality 20), and reads that aligned against the CHM13 v2 complete human reference (GenBank: GCA_009914755.4) using minimap2 (v2.17‐r941) were removed.^42^, ^43^, ^44^ Consensus sequences were generated using minimap2 (v2.17‐r941), samtools (v1.15) and iVar (v1.3.2) with a 75% variant frequency threshold, and a minimum of 20 reads per site, against the US‐CDC outbreak reference, MPV_USA_2022_MA001 (GenBank accession: ON563414.3)^44^, ^45^, ^46^ to call major variants. For samples with Oxford Nanopore Technology (ONT) reads only, read quality control was assessed using using nanoq (v0.9.0).^47^ Consensus sequences were generated with Medaka (v1.6.1, r941_min_hac_variant_g507, https://github.com/nanoporetech/medaka) with a minimum of 20 reads per site, against the US_CDC outbreak reference, MPV_USA_2022_MA001 (GenBank accession: ON563414.3) to call major variants. Consensus genomes were included in further analyses if they contained ≥90% non‐N characters, relative to the reference genome length for Illumina samples, or ≥85% non‐N for ONT samples. Nextflow pipelines with settings used here and dependencies for both Illumina and ONT reads are available (https://github.com/vidrl/mpx-vidrl).^48^

### 4.4 Major and minor variant calling

Major variants were defined as those present in the consensus genomes by either iVar (v1.3.2) (≥75% frequency for samples sequenced on an Illumina platform), or Medaka (v1.6.1) (for samples sequenced on an ONT platform).^46^ For samples sequenced on an Illumina platform, analysis of minor variants at a frequency between 5% and 75% was performed using iVar (v1.3.2), and output using a custom script.^46^ In all samples, major and minor variants were not considered in repeat regions defined by Tandem Repeats Finder (v4.09) or in the terminal inverted repeat region of the genome (Supporting Information: Table 1).^49^ The sequence context flanking all detected SNPs was determined using a python script developed by Isidro et al.,^11^ to screen whether SNPs follow signatures potentially compatible with APOBEC3‐mediated viral genome editing (namely, GA  >  AA and TC  >  TT replacements) (https://github.com/insapathogenomics/mutation_profile). Variants and nucleotide positions are always relative to the US‐CDC outbreak reference (MPV_USA_2022_MA001, GenBank accession: ON563414.3), approximating the B.1 ancestor.

### 4.5 Lineage designation

Lineage designation for sequences was determined using Nextclade (v2.8.0) using the hMPXV database, downloaded on October 31, 2022, with confirmation by manual inspection of the variant sites. Positional coordinates of lineage defining SNPs are reported in text after manual conversion from reference NC_063383 (GenBank accession: MPV‐M5312_HM12_Rivers) to the US_CDC outbreak reference MPV_USA_2022_MA001 (GenBank accession: ON563414.3) (Table 2).^50^

**Table 2:** MPXV lineage defining variants.

Lineage	Nucleotide position (NC_063383)	Nucleotide position (ON563414.3)	ALT allele	Gene (ON562414.3)	Vaccinia virus homolog	Change	APOBEC3
B.1	77 383	77 400	A	*MPXVgp083*	*LR4*	NONSYN	GA ‐ > AA
B.1.1	74 360	74 377	A	*MPXVgp079*	*GR9*	NONSYN	NO
B.1.2	186 165	186 144	A	*MPXVgp182*	‐	NONSYN	GA ‐ > AA
B.1.3	190 660	190 639	A	*MPXVgp187*	‐	NONSYN	GA ‐ > AA
B.1.4	34 308	34 325	A	*MPXVgp041*	*F9L*	SYN	GA ‐ > AA
B.1.5	70 780	70 797	T	*MPXVgp074*	*G5R*	NONSYN	TC ‐ > TT
B.1.6	111 029	111 046	A	*NON CDS*	‐	NA	GA ‐ > AA
B.1.7	25 644	25 660	T	*MPXVgp029*	*K3L*	NONSYN	TC ‐ > TT
B.1.8	5595	5612	A	*MPXVgp004*	‐	SYN	GA ‐ > AA
B.1.9	181 367	181 346	A	*MPXVgp182*	‐	SYN	GA ‐ > AA
B.1.10	94 798	94 815	A	*MPXVgp100*	*D3R*	NONSYN	GA ‐ > AA
B.1.10	89 906	89 923	T	*MPXVgp095*	*H5R*	NONSYN	TC ‐ > TT
B.1.11	159 277	159 270	A	*MPXVgp163*	*A56R*	NONSYN	GA ‐ > AA
B.1.11	18 133	18 150	T	*MPXVgp016*	*C4L*	NONSYN	TC ‐ > TT
B.1.12	182 950	182 929	T	*MPXVgp182*	‐	NONSYN	TC ‐ > TT
B.1.13	175 093	175 090	A	*MPXVgp177*	*B19R*	NONSYN	GA ‐ > AA
B.1.14	36 617	36 634	A	*MPXVgp043*	*F11L*	SYN	GA ‐ > AA
B.1.14	159 779	159 772	T	*MPXVgp163*	*A56R*	NONSYN	TC ‐ > TT

Abbreviation: MPXV, monkeypox virus.

### 4.6 Assessing large‐scale genomic changes

Read depth was calculated at each site and using a sliding window across the reference genome (MPV_USA_2022_MA001, GenBank ON563414.3). Continuous regions >500 bps with a read depth of 2× the mean read depth per sample were further investigated for genomic duplications. Continuous regions >500 bps with a read depth of 0 were further investigated for genomic deletions. Read mapping profiles suggesting a putative deletion in the genome were further investigated by using minimap2 (v2.17‐r941) to align reads to an edited version of the reference genome (MPV_USA_2022_MA001, GenBank ON563414.3) in which the region of a suspected deletion was manually removed.^44^ Reads were also mapped using minimap2 (v2.17‐r941) to a sequence of the suspected deletion.^44^ Sanger sequencing of the suspected deletions junctions was used to additionally confirm the deletions. PCR was used to amplify the suspected deletion regions using custom oligonucleotides (Supporting Information: Table 2). Amplicons were purified using a QIAquick PCR purification kit (Qiagen) according to the manufacturer's instructions and sequenced using standard Sanger sequencing methods using the appropriate oligonucleotides (Supporting Information: Table 2) by the Victorian Clinical Genetics Service.

### 4.7 Phylogenetic analyses

Publicly available MPXV consensus genomes from The National Center for Biotechnology Information (NCBI) or Nextstrain were accessed on October 31, 2022.^13^ A subset of these genomes, meeting the inclusion criteria (>182 000 bp, containing ≥90% non‐N characters, collected between 2018 and 2022, lineages A or B, and had comprehensive accompanying metadata) to a total of 1003 genomes were used in combination with the Victorian samples to make an alignment using the Nextclade aligner (v2.8.0) (Supporting Information: Data set 1).^50^ A ML phylogenetic tree was made using Iqtree2 (v2.0.3) using 1000 ultrafast bootstraps and the HKY + F + I model allowing for polytomy.^51^ Pairwise SNP distances between genomes were calculated with snp‐dists (v0.8.2, https://github.com/tseemann/snp-dists). To confirm the geographical distribution of selected SNPs, all remaining MPXV genomes (*n* = 2190) on NCBI were accessed on October 31, 2022.

### 4.8 Data visualization

Visualization of variant sites from sequence alignments for individual individuals was performed using Snipit (https://github.com/aineniamh/snipit). Visualization of read pileup was performed using Geneious Prime All other plots were generated with ggplot2 (v3.4.0) and ggtree (v.3.6.2). All statistical analyses were performed in Rstudio (v4.2.2).

## AUTHOR CONTRIBUTIONS

Deborah A. Williamson, Leon Caly, and Mona L. Taouk conceived and designed the study. Ivana Savic and George Taiaroa prepared genomic libraries and performed sequencing. Thomas Tran performed quantitative polymerase chain reaction. Eike Steinig, Mona L. Taouk, and George Taiaroa performed the bioinformatic analyses. Nasra Higgins, Alvin Lee, and Maxwell Braddick provided patient metadata. Mona L. Taouk prepared the figures. Draft publication was written by Mona L. Taouk, George Taiaroa, Eike Steinig, and Deborah A. Williamson, and was reviewed by all authors.

## CONFLICT OF INTEREST STATEMENT

The authors declare no conflict of interest.

## ETHICS STATEMENT

Ethics was sought and obtained from Alfred Ethics (505/22).

## Supporting information

Supporting Information.

Supporting Information.

Supporting Information.

## Data Availability

The data that support the findings of this study are openly available in GenBank at https://www.ncbi.nlm.nih.gov/genome/. Consensus genomes generated in this study and included in analyses are deposited in GenBank under accessions OR264364 – 465 (Supporting Information: Data set 1).
