# Supplementary material for: Intra‐ and interhost genomic diversity of monkeypox virus
Source: J Med Virol. 2023 Aug 11;95(8):e29029. doi: 10.1002/jmv.29029 (PMC10952654; doi:10.1002/jmv.29029)
Supplement: Supplementary file 3 — Supporting Information. [file JMV-95-0-s003.docx]

SUPPLEMENTARY APPENDIX

Taouk et al.

Intra- and inter-host genomic diversity of monkeypox virus

[Supplementary Results 3](#_Toc140687773)

[Quality control of consensus genomes 3](#_Toc140687774)

[Comparison of genomes generated from Illumina reads and genomes generated from Oxford Nanopore reads 3](#_Toc140687775)

[Further examples of discordance between lineage-defining SNPs and phylogenetic clades 3](#_Toc140687776)

[Diversity in Victorian MPXV genomes may include large scale multi-gene duplications 4](#_Toc140687777)

[Supplementary Figures 5](#_Toc140687778)

[Supplementary Figure 1 5](#_Toc140687779)

[Supplementary Figure 2 6](#_Toc140687780)

[Supplementary Figure 3 8](#_Toc140687781)

[Supplementary Figure 4 9](#_Toc140687782)

[Supplementary Figure 5 10](#_Toc140687783)

[Supplementary Tables 11](#_Toc140687784)

[Table 1: Regions excluded from variant analyses 11](#_Toc140687785)

[Table 2: Primers used for PCR and Sanger sequencing 12](#_Toc140687786)

[Description of Supplementary Datasets 13](#_Toc140687787)

[Supplementary Dataset 1 13](#_Toc140687788)

[Supplementary Dataset 2 13](#_Toc140687789)

# Supplementary Results

## Quality control of consensus genomes

Following quality control measures, 50/80 (62.5%) of consensus genomes generated from Illumina reads and 52/115 (46.4%) of consensus genomes generated from ONT reads were included in these analyses to a total of 102 genomes (Supplementary Figure 1c).

## Comparison of genomes generated from Illumina reads and genomes generated from Oxford Nanopore reads

For 19 samples, both Twist and Midnight sequencing libraries were generated and these samples were sequenced using both the Illumina platform and the ONT method. 3/19 passed quality control for both platforms. Consensus genomes were generated as described in the methods for these three pairs of samples, and the genomes aligned. It was found that there were no SNP differences between the sample sequenced on Illumina or the sample sequenced on ONT for any of the three pairs of genomes.

## Further examples of discordance between lineage-defining SNPs and phylogenetic clades

A monkeypox virus genome (accession: OP605562, sequenced in Slovenia in October 2022) containing the B.1.3 lineage defining SNP (G to A at nucleotide position 190,639) was positioned outside of the B.1.3 clade in the phylogeny amongst the other B.1 samples. Further, there was evidence of genomes containing the lineage defining variant for more than one lineage. For example, ON637939, collected from a patient in Germany in May 2022, has the variants required to be defined as both B.1.5 and B.1.7. However, this genome was clustered within the B.1.7 clade in the phylogeny. Additionally, there were two instances of genomes (accessions: OP415208, OP415209; sequenced in the UK in September 2022) containing the B.1.2 lineage defining SNP (G to A at nucleotide position 186,144) positioned outside the B.1.2 clade in the phylogeny amongst other B.1 genomes, indicating that the genomes acquired the mutation independently of the B.1.2 common ancestor.

Further, there is evidence of genomes containing the lineage defining variant for more than one lineage within the Victorian dataset. Two genomes (34_A, 34_B), had the B.1.5 lineage defining SNP (C70797T) as well as the B.1.2 lineage defining SNP (G186144A), but were clustered in the B.1.2 clade of the phylogeny. When removing the B.1.2 SNP from the alignment, these samples clustered within the B.1.5 lineage clade (Supplementary Figure 4). Additionally, in this phylogeny excluding the B.1.2 lineage defining SNP, the genomes containing the B.1.2 variant were scattered throughout, with the most common clade being B.1. Interestingly, the B.1.2 lineage defining mutation results in the amino acid substitution D1604N in the surface glycoprotein OPG210, as well as being a target for APOBEC3 activity (GA to AA).

## Diversity in Victorian MPXV genomes may include large scale multi-gene duplications

In addition to the well characterised deletions observed in this dataset, we found evidence of a large scale genomic duplication in one sample (51_B), which was the only sample collected from individual 51. A region of approximately 25,150 bp in the 3’ region of the genome (nucleotide positions 164,950 to 190,100) was observed to have double the average read depth compared to the rest of the genome, suggestive of a duplication event (Supplementary Figure 5).

# Supplementary Figures

## Supplementary Figure 1


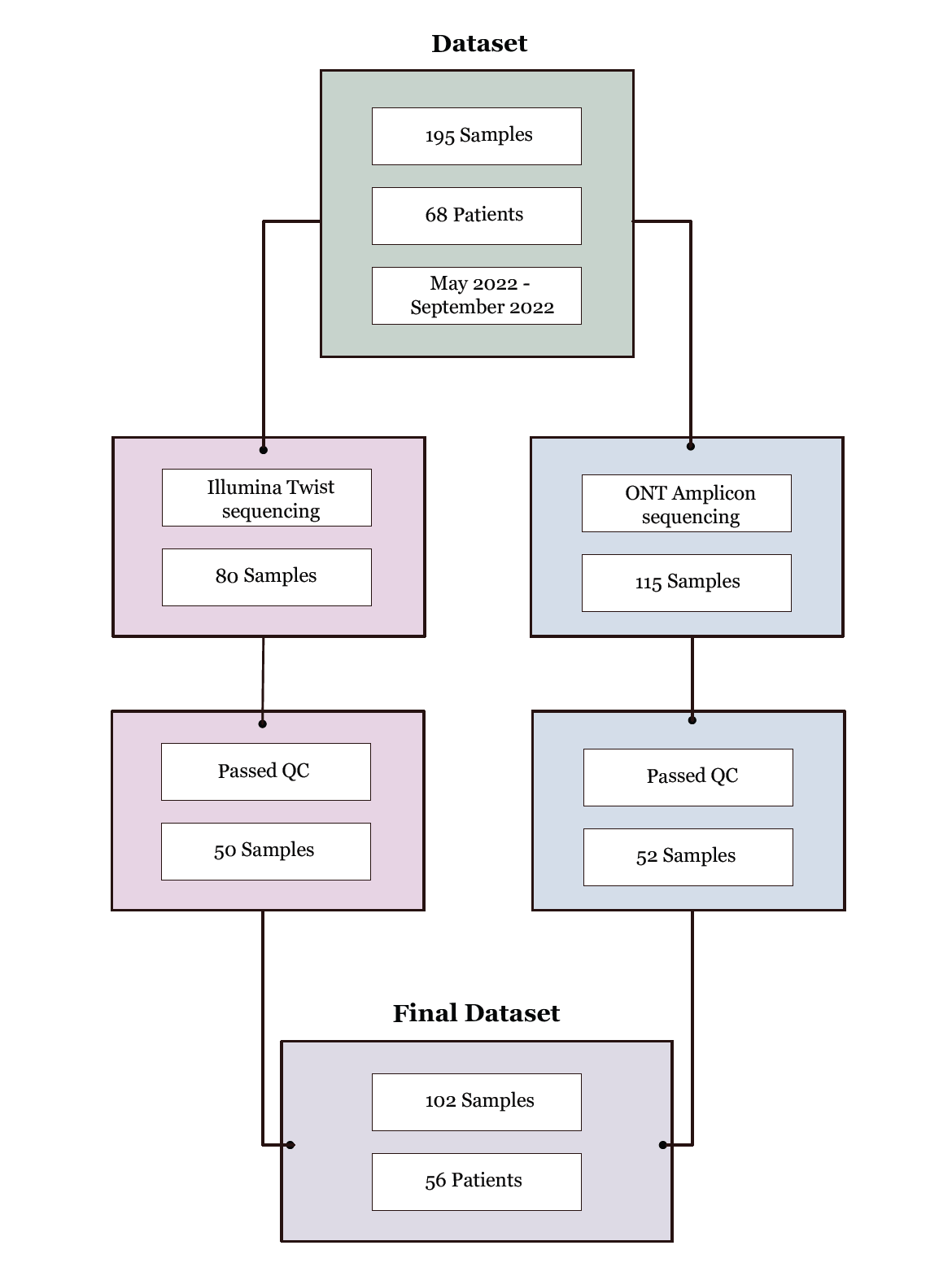


A flow chart showing the number of consensus genomes included in phylogenetic analyses from each sequencing platform (Oxford Nanopore Amplicon sequencing or Illumina Twist enrichment sequencing).

### Supplementary Figure 2


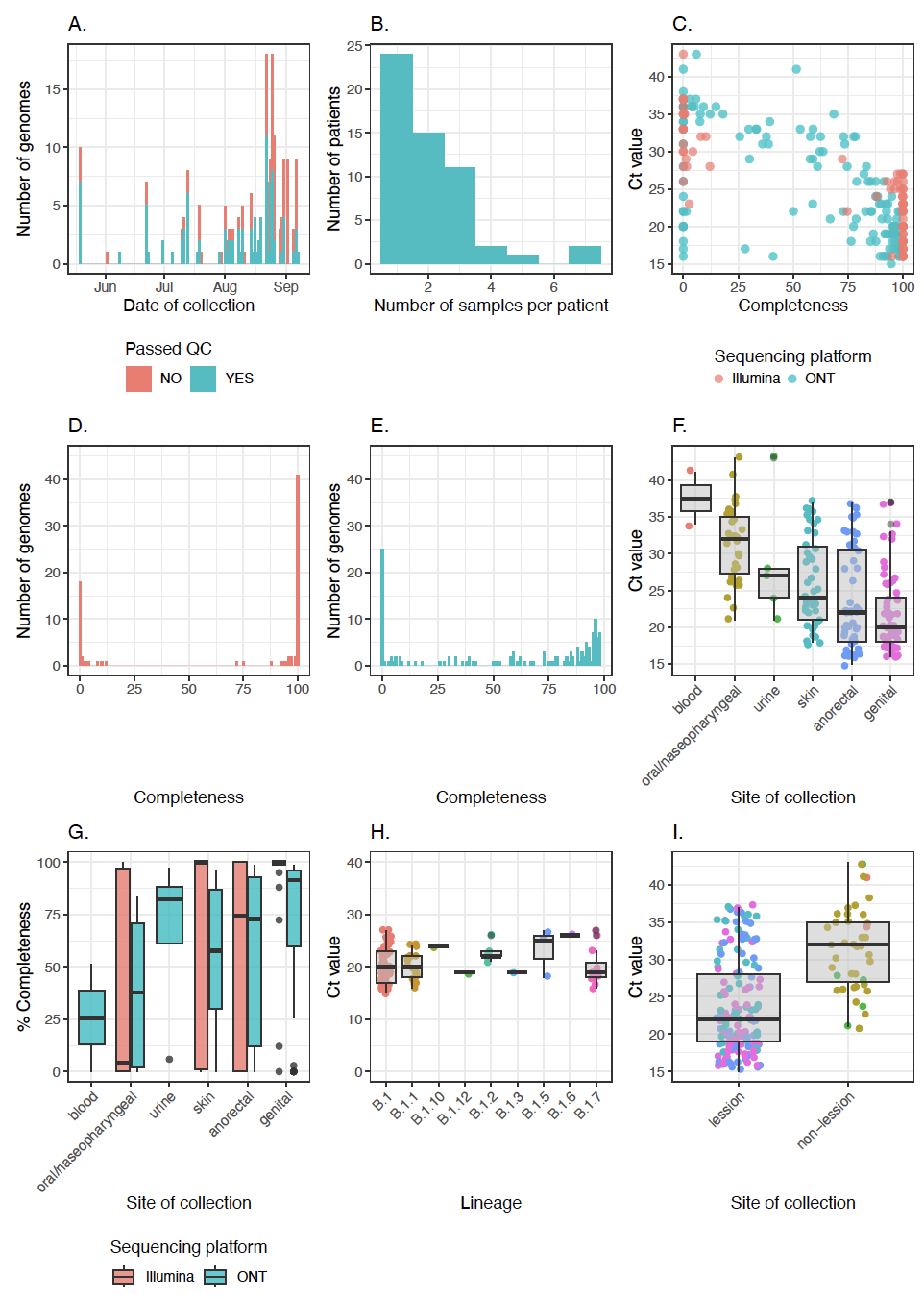


**A.** Distribution of dates of sample collection coloured by whether the genome passed QC and was included in the final analyses. **B.** Histogram showing the distribution of number of samples collected per patient, only including genomes included in the final analyses. **C.** Ct value plotted against genome completeness (percentage of non-N characters per consensus genome) for each sample sequenced. **D.** Distribution of genome completeness for samples sequenced on an Illumina device. **E.** Distribution of genome completeness for samples sequenced on an ONT device. **F.** Ct value plotted by site of sample collection. Box plots indicate median and interquartile range (IQR), with the whiskers representing the highest and lowest values within 1.5 × IQR of the upper and lower quartiles. **G.** Genome completeness plotted by site of sample collection and coloured by sequencing platform method. **H.** Ct value plotted for each sample by lineage designation. **I.** Ct value plotted by site of sample collection, summarised by lesion samples (skin, anorectal, genital) or non-lesion samples (oral/nasopharyngeal, blood, urine).

### Supplementary Figure 3


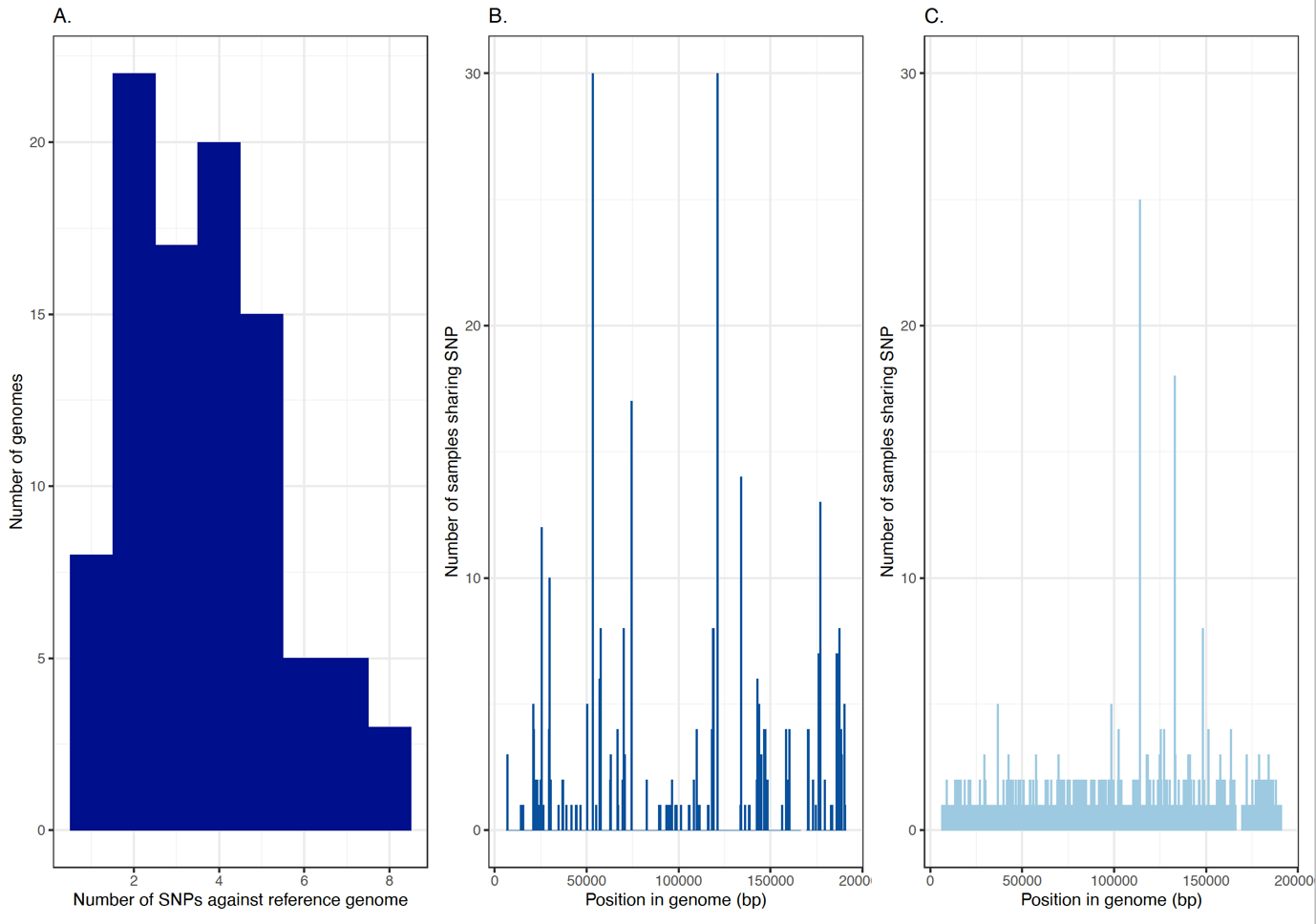


**A.** A histogram showing the number of SNPs per consensus genome against the US B.1 reference (MPV_USA_2022_MA001, GenBank ON563414.3). **B.** The number of major SNPs in each unique position of the reference genome (MPV_USA_2022_MA001, GenBank ON563414.3). **C.** The number of minor SNPs in each unique position of the reference genome (MPV_USA_2022_MA001, GenBank ON563414.3).

### Supplementary Figure 4


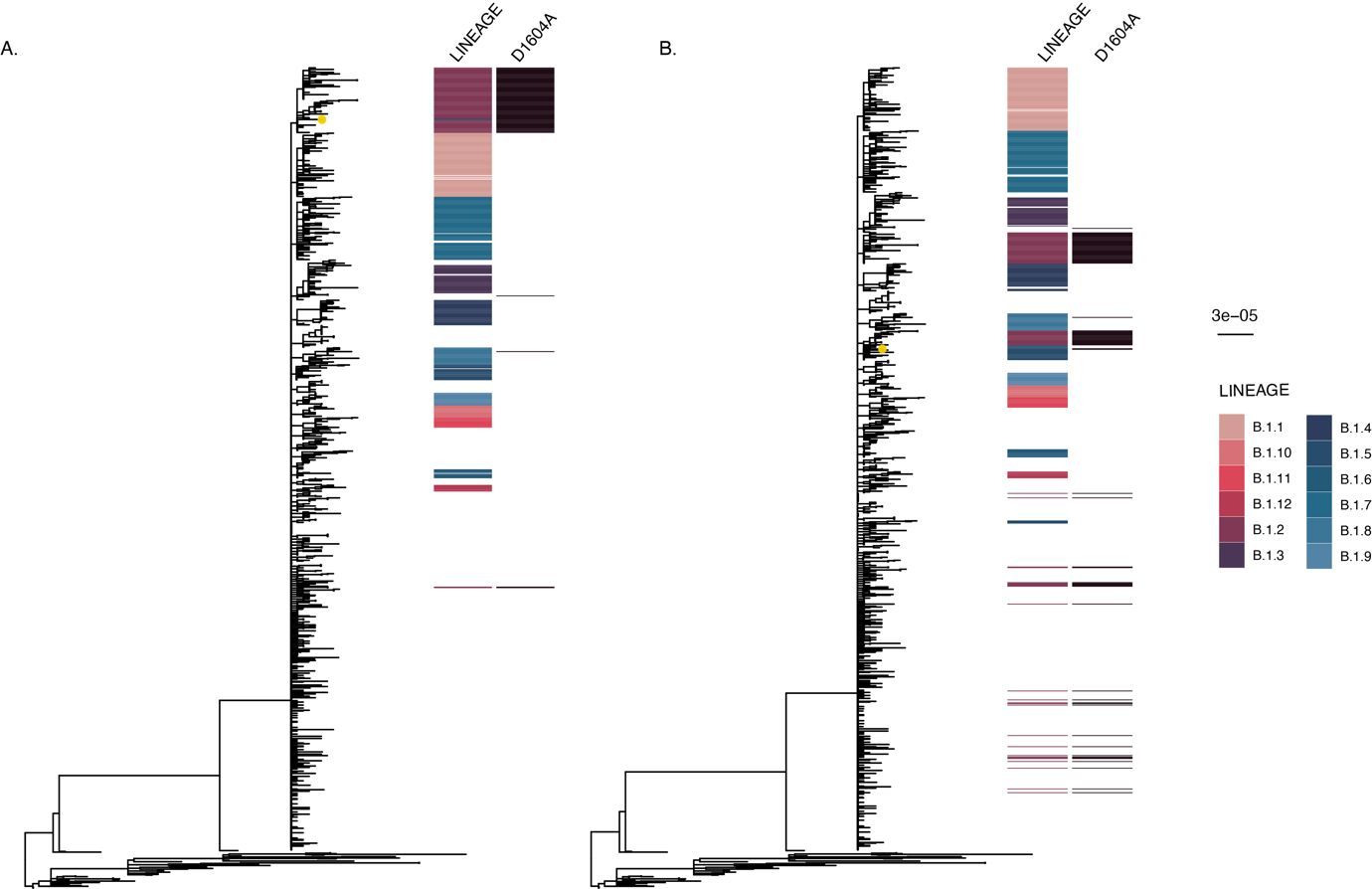


**A.** A midpoint rooted maximum likelihood phylogeny showing the population structure of 1103 MPX genomes included in this study, using the whole genome alignment with 607 parsimony-informative sites, 988 singleton sites and 195614 constant sites. Next clade lineages B.1.1-B.1.12 are plotted as a heatmap. Genomes containing the B.1.2 lineage defining SNP are also plotted as a heatmap. The two genomes in this study containing both the B.1.2 and B.1.5 lineage defining SNP are coloured by a yellow dot on their tips. The scale represents substitutions per site. **B.** A midpoint rooted maximum likelihood phylogeny, using the whole genome alignment with the B.1.2 lineage defining SNP (G186144A) removed. Next clade lineages B.1.1-B.1.12 are plotted as a heatmap. Genomes containing the B.1.2 lineage defining SNP are also plotted as a heatmap. The two genomes in this study containing both the B.1.2 and B.1.5 lineage defining SNP are coloured by a yellow dot on their tips.

### Supplementary Figure 5


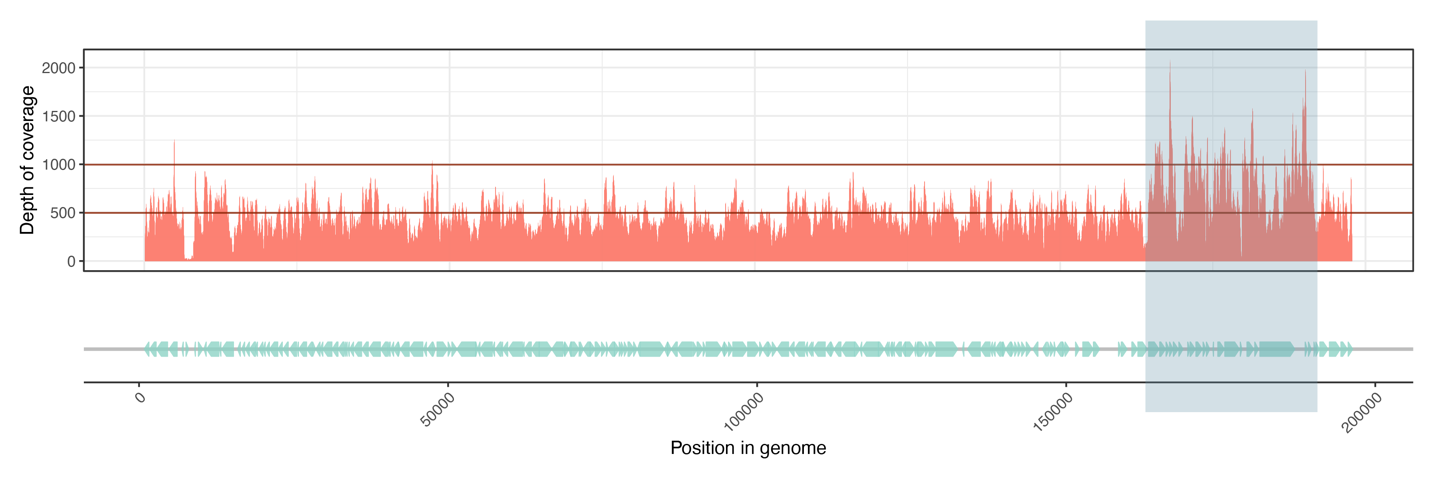


Reads from sample 51_B assembled to the MPV_USA_2022_MA001 (GenBank: ON563414.3) reference genome with genome positions (base pairs) on the X axis including positions and directions of genes. Coverage, or number of aligned reads, are plotted on the Y axis. The region of a suspected duplication is highlighted by the grey rectangle. The mean read depth is represented by the red line. 2x the mean read depth is represented by the red line.

# Supplementary Tables

## Table 1: Regions excluded from variant analyses

| **Reference** | **Start** | **End** | **Description** |
| --- | --- | --- | --- |
| ON563414.3 | 1 | 6438 | Terminal repeat |
| ON563414.3 | 133095 | 133122 | Homopolymer |
| ON563414.3 | 136524 | 136593 | Tandem repeat |
| ON563414.3 | 140112 | 140201 | Tandem repeat |
| ON563414.3 | 141987 | 142032 | Tandem repeat |
| ON563414.3 | 146850 | 146923 | Tandem repeat |
| ON563414.3 | 150550 | 150622 | Tandem repeat |
| ON563414.3 | 163184 | 163216 | Tandem repeat |
| ON563414.3 | 166085 | 166110 | Tandem repeat |
| ON563414.3 | 169716 | 169768 | Tandem repeat |
| ON563414.3 | 173268 | 173315 | Tandem repeat |
| ON563414.3 | 174517 | 174545 | Tandem repeat |
| ON563414.3 | 179054 | 179223 | Tandem repeat |
| ON563414.3 | 190765 | 197205 | Terminal repeat |

### Table 2: Primers used for PCR and Sanger sequencing

| **Sequence (5'-3')** | **Description** |
| --- | --- |
| CCAACAACGGACCACATCCT | Internal to patient 48 deletion |
| TCTACATGCGTATTGTGGCA | Internal to patient 48 deletion |
| ACCGTCGGATAGCGTCATTC | External to patient 48 deletion |
| AGCGTGTTCAGTATCCGCA | External to patient 48 deletion |
| ACCAACACAAAATTCGACCGT | Internal to patient 34 deletion |
| CACCCAACTGTTTAGCGTGC | Internal to patient 34 deletion |
| AGCCACAGAAGGGTTTCTCG | External to patient 34 deletion |
| GGGGCGAATACTCTTCCCAA | External to patient 34 deletion |

# Description of Supplementary Datasets

## Supplementary Dataset 1

Sheet 1: Metadata for all genomes generated in this study and all previously published genomes included in the phylogenetic analyses.

## Supplementary Dataset 2

Sheet 1: Description and positions of major variants

Sheet 2: Description and positions of minor variants
